# Flexible Electrowetting-on-Dielectric Microlens Array Sheet

**DOI:** 10.3390/mi10070464

**Published:** 2019-07-11

**Authors:** Kari L. Van Grinsven, Alireza Ousati Ashtiani, Hongrui Jiang

**Affiliations:** 1 Department of Electrical and Computer Engineering, University of Wisconsin-Madison, Madison, WI 53706, USA; 2 Department of Materials Science and Engineering, University of Wisconsin-Madison, Madison, WI 53706, USA; 3 Department of Ophthalmology and Visual Sciences, University of Wisconsin-Madison, Madison, WI 53706, USA; 4 McPherson Eye Research Institute, University of Wisconsin-Madison, Madison, WI 53706, USA

**Keywords:** electrowetting-on-dielectric, flexible, tunable liquid lens, large field of view, tunable lens array

## Abstract

We have fabricated a fully-flexible, focus-tunable microlens array on a sheet and demonstrated its imaging capabilities. Each liquid lens of the array is individually tunable via electrowetting on dielectric (EWOD) actuation and is situated on a polydimethylsiloxane (PDMS) substrate, which allows the lens array to operate as a reconfigurable optical system. In particular, we observed a significant increase in the field of view (FOV) of the system to 40.4° by wrapping it on a cylindrical surface as compared to the FOV of 21.5° obtained by the array on a planer surface. We also characterized the liquid lenses of the system, observing a range of focus length from 20.2 mm to 9.2 mm as increased voltage was applied to each EWOD lens. A Shack–Hartmann wavefront sensor (SHWS) was used to measure the wavefront of the lens as it was actuated, and the aberrations of the lens were assessed by reporting the Zernike coefficients of the wavefronts.

## 1. Introduction

Liquid microlenses have been well established as viable solutions to enable tunable focal lengths for imaging, leading to increases in versatility of miniature imaging systems. Tunable lenses operate by either changing the refractive index of the lens or by changing its curvature in order to induce a change in the focal length. They usually utilize two liquids of different indices of refraction, the curved interface between them defining the lens. There are a large variety of actuation methods that have been demonstrated for liquid lenses, including fluidic pressure [1,2,3], liquid crystal [4,5], hydrogel [6,7], thermal [8,9], dielectrophoretic [10,11], and electrowetting [12,13,14,15]. Electrowetting is particularly attractive thanks to its fast response times [16,17] and low power consumption. Additionally, since we plan to fabricate an array of lenses, it is relatively simple to electrically insulate neighboring electrowetting lenses from one another, something not easily implemented in, for example, thermally actuated lenses.

While microlenses were typically fabricated on rigid structures, there has been significant work in recent years to construct tunable liquid lenses on flexible substrates [18,19,20,21]. Similarly, there has been a variety of research on tunable microlens arrays [22,23,24,25,26,27]. However, the vast majority of such arrays have been either on rigid substrates or have shared actuation between lenses, meaning the focal length of one lens in the array cannot be tuned independently from its neighbor. Finally, almost none of these microlens array seek to image far-field scenes.

By seeking to create an array of electrowetting lenses on a flexible substrate we endeavor to achieve a highly reconfigurable imaging platform for visible wavelengths, capable of providing a wide field of view (FOV) when wrapped on a convex surface. This is done by stitching the individual images from the lenses in the array together into a single image, and the extent of the increase in FOV will be determined by the radius of curvature of the underlying surface. It is only limited by the requirement that there is at least some overlap between each image formed by the array. The independent turning of each lens in the array may also allow for imaging with a greater depth of field. These capabilities in combination with the unobtrusive form factor of the microlens array could have particularly relevant applications in imaging for monitoring or surveillance purposes. Additionally, wrapping the flexible substrate on a concave surface would enable the array to also be utilized for 3D imaging. In order to achieve the high yield required to make use of an array in which the entire array is fabricated together and individual components cannot be exchanged, we made use of an areal density modulated electrode design that we previously reported [15], which simplified the fabrication process and improved yield.

## 2. Materials and Methods 

### 2.1. Principles of Operation and Design

**Figure 1:**
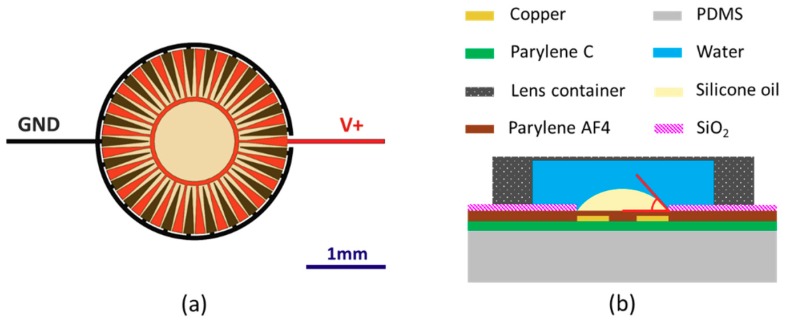
(**a**) Top-down view of areal-density-modulated electrode design, where live and grounded electrodes are interdigitated. The pale, yellow disk over the center of the electrodes highlights the area where the surface is hydrophobic, where the oil will initially pin. As voltage is applied the oil droplet will be symmetrically squeezed by the water which surrounds it. (**b**) Cross-section of a schematic of a single, fully assembled electrowetting lens (not to scale). The well for containing the liquids for electrowetting was affixed with adhesive, and a silicone oil droplet was placed over the hydrophobic region of the flexible dielectric surface defined by the patterned SiO_2_ before being surrounded by water and sealed into the well. The red angle indicates the contact angle of the oil droplet.

Our actuation mechanism makes use of an areal density modulated electrode design with which we previously demonstrated electrowetting on dielectric (EWOD) functionality. While previous work focused on characterizing a single EWOD lens on a rigid substrate [15], or on developing methods to fabricate EWOD devices on flexible substrates [16], in this work we demonstrated a completely functional tunable lens array utilizing EWOD, and also turned our attention to the imaging capabilities of an array of lenses, making improvements to the design and fabrication methods in the process. The areal-density-modulated electrode design used by each liquid lens in our array can be seen in Figure 1a. It shows a circular grouping of interdigitated electrodes across which we apply voltage. The dielectric in our EWOD lenses is a relatively new kind of Parylene, a fluorinated Parylene known as Parylene AF4, which is hydrophobic. We add a thin, hydrophilic layer of SiO_2_ to the surface surrounding our interdigitated electrodes in order to define a rest state for the oil droplet and surrounding water that composes our lens. As seen in Figure 1b the silicone oil droplet (with a refractive index of 1.49) would naturally pin at the Parylene AF4/ SiO_2_ boundary as its rest state, creating a convex lens. More detail on material choices and fabrication methods can be found in Section 2.2. 

This design has several benefits, the first of which is that it allows analog focal length tuning of our lenses. As increasing voltage is applied across the underlying electrodes, the water of the lens—which acts as a third, floating electrode—is attracted down and in towards the electrodes, effectively squeezing the oil droplet. This squeezing means that the surface area covered by the oil droplet will decrease, and since the overall volume of oil must remain unchanged, the contact angle of the droplet (which forms a spherical cap) increases. This increase in contact angle corresponds to a decrease in the radius of curvature of the lens, effectively shortening the focal length of the system. The initial contact angle of the lens is determined by the volume of its silicone oil droplet, and the contact angle of the lens as a function of the applied voltage is described by Equation (1):

(1) $$\cos\theta \left(V\right)=\cos\theta_{0}-\frac{\varepsilon_{0}\varepsilon_{r}}{2d\gamma }V^{2}$$

where θ(V) is the contact angle of the oil droplet as a function of voltage, $\theta_{0}$ is the initial contact angle (zero volts applied), $\varepsilon_{r}$ is the relative permittivity of the dielectric layer, d is the thickness of the dielectric, γ is the interfacial surface tension of the oil and water, and V is the voltage applied. It should be noted that the sign of the equation is inverted compared to the standard electrowetting equation to account for the switching of the physical positions of the conductive liquid (here, water) and the insulating liquid (here, silicone oil) [28]. The superior film quality we achieved with Parylene AF4 (Yuan-Shin Materials Technology Corp., Kaohsiung, Taiwan) made it possible for us to significantly reduce the thickness of the dielectric layers, which reduced the required driving voltages.

The areal-density-modulated electrodes also serve as a self-centering mechanism for the lens, to ensure it remains centered on its optical axis. This effect is achieved by taking advantage of the fact that the system that composes our lenses will always attempt to minimize its potential energy. In order to do so, the capacitance of the system must be maximized, according to equation (2):

(2) $$U=\frac{1}{2}\frac{q^{2}}{C},$$

where U is the potential energy, q is the stored charge, and C is the capacitance. Our electrodes have been designed such that the percentage of surface area where electrodes are present varies as a function of the radius from the central point of the electrodes. As seen in Figure 1a, at their outer edge (i.e., largest radial distance) the interdigitated electrodes cover the overwhelming fraction of the circumference, and as the radial distance decreases the electrodes cover a decreasing fraction of surface area. In order for the system to maximize the capacitance, the water and oil will move so the water covers the largest possible area of the electrodes. This can only be achieved when it is centered over the electrodes, thus fixing the optical axis of the system [15]. This centering feature of the areal-density-modulated electrodes enables us to use a wholly planer design for our EWOD lens, which greatly simplifies the fabrication and improves the yield, the latter of which is crucial in the creation of an array of lenses.

### 2.2. Fabrication

**Figure 2:**
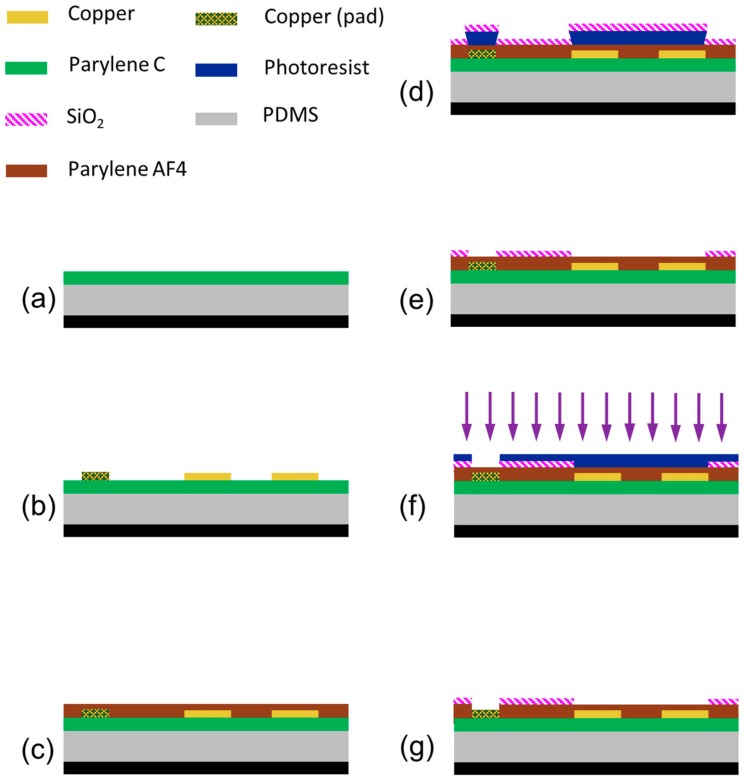
Microfabrication process of a flexible electrowetting-based tunable lens array. (**a**) 250 µm of PDMS is spin coated onto a carrier wafer. After PDMS is cured, a 2-µm layer of Parylene C is deposited as an adhesive layer. (**b**) 275 nm of copper is deposited via magnetron sputtering and then patterned and wet-etched into areal density modulated array electrodes along with traces and pads. (**c**) 800 nm of Parylene AF4 is deposited over the whole wafer to act as a pinhole free dielectric layer. (**d**) A lift-off type photoresist is patterned to cover contact pads and define the hydrophobic region immediately over the areal-density-modulated electrodes for each lens, then 100 nm of SiO_2_ is deposited via radio-frequency (RF) sputtering. (**e**) Lift-off of SiO_2_ was preformed via ultrasonic agitation in acetone. (**f**) Positive photoresist is patterned to protect all of wafer except areas over contact pads. A 200-W oxygen plasma is applied to etch Parylene AF4 down to copper contact pads to allow electrical contact to each device in the array. (**g**) Photoresist is stripped and a final cleaning of the wafer is completed.

In order to create our reconfigurable microlens array sheet we require the sheet to be suitably flexible while robust, and all materials must be chosen accordingly. The fabrication process, which needed to take into account the specific challenges inherent in working with such flexible materials, is shown in Figure 2. It began with the use of a carrier wafer (3 inch, mechanical grade silicon) that supported our polydimethylsiloxane (PDMS) substrate. The PDMS (SYLGARD 184, Dow, Midland, MI, USA) was deposited via spin coating at 500 rpms for 30 s. The edge bead which results from the spin coating process was removed manually, and then the PDMS was allowed to self-planarize on a level surface at room temperature before it was cured in a 75 °C oven for 7 h. This yielded a 250-µm PDMS layer, which is thin enough to give us a large degree of flexibility, but thick enough to be easily handled without damaging it. The wafer was then coated with a 2-µm layer of Parylene C in order to improve adhesion in the following sputtering steps, and to increase the stability of the flexible substrate. 

Next, a copper thin film of 275 nm was deposited onto the PDMS and Parylene C substrate via magnetron sputtering. The sputtering was performed at a low power of 200 W in order to reduce thermal expansion of the PDMS. After sputtering, the electrodes for each microlens in the array, their traces and contacting pads were all patterned via contact photolithography. Positive photoresist (S1813, MicroChem Corp., Westborough, MA, USA) was spin coated, exposed, developed, rinsed, and finally hard baked so that the photoresist could be used as a mask for wet etching of the copper. Wet etching was performed using a commercial copper etchant (APS-100 copper etchant, Transene Company, Danvers, MA, USA) diluted with DI water in a 4 to 1 ratio (DI water to etchant) and was done by submerging the wafer for 3 min at room temperature. The photoresist was then stripped, and the wafer rinsed prior to deposition of our dielectric layer. 

We chose as our dielectric Parylene AF4 (sometimes referred to as Parylene HT), a fluorinated Parylene. While all variants of Parylene films have the attractive qualities of providing a conformal coating, being flexible, and providing good optical transparency in the visible range, Parylene AF4 has a number of unique characteristics that make it particularly attractive for use in our design. Many EWOD actuators have a prerequisite dielectric material, and then add another hydrophobic layer (usually an amorphous fluoropolymer such as Teflon). Nevertheless, since Parylene AF4 is inherently hydrophobic (contact angle of ~100°), an additional layer is not required. This allows for a decrease in the overall dielectric thickness, which reduces required driving voltages. Parylene AF4 also has superior penetration depths (up to 50:1 aspect ratio), which leads to superior film quality compared to the more common Parylene C [29]. The Parylene AF4 film was deposited using a vacuum vapor deposition polymerization (VDP) process in a LabTop 3000 Parylene coating system (Para Tech Coating, Inc., Aliso Viejo, CA, USA) with Parylene AF4 dimer as the raw material for the system. As the Parylene moves through the coating system, the Parylene AF4 dimer is first vaporized, then broken down into a monomer in the pyrolysis chamber (heated to 705 °C) and finally polymerizes as a thin film onto our wafers in the deposition chamber at room temperature [30]. The resulting film was an 800 nm conformal coating of Parylene AF4 (see Figure 2c), which served as the insulating, dielectric layer to prevent shorts from occurring in our electrowetting devices. The wafer was coated in the dielectric material (dielectric constant of 2.25) with a robust breakdown voltage (216 kV/mm) [31]. The one drawback of Parylene AF4 is that, like most fluorinated materials it has a relatively low dielectric constant (which decreases the potential change in contact angle as seen in Equation (1)). However, the dramatic reduction in thickness allowed by the superior film quality more than compensates for this decrease.

After Parylene AF4 deposition, the entire wafer has been electrically insulated by the hydrophobic material. In order to help pin the liquids that our tunable liquid lenses will be composed from, we need the surface surrounding the underlying interdigitated electrodes to be hydrophilic, while the area directly over the electrodes remains hydrophobic. This circular disk with hydrophobic surface properties will define where our oil droplet resides and allows the surrounding water to naturally pin at the boundary (see Figure 1b). While all Parylene, including Parylene AF4, can be rendered temporarily hydrophilic via treatment with an oxygen plasma, we instead chose the more permanent solution of depositing a thin SiO_2_ layer in the regions that require a hydrophilic surface property. The SiO_2_ was deposited via radio-frequency (RF) sputtering in conjunction with a lift-off process to pattern, as seen in Figure 2d. This lift-process involved several steps. First, a negative lift-off photoresist (KL1604, KemLab, Inc., Woburn, MA, USA) was patterned via photolithography to protect the circular disks for our lens placement as well as the areas over the contact pads. The wafer was then deposited with 100 nm of SiO_2_ via RF sputtering at 140 W. After sputtering, the portions of film that had been deposited on the photoresist was removed by transferring the wafer to a beaker of acetone, which was then placed in an ultrasonic bath until the lift-off resist and the corresponding portions of the SiO_2_ film was removed, as shown in Figure 2e. 

Next, in order to gain access to the contact pads on the edges of the wafer where external voltage will be applied to the electrowetting devices, the Parylene AF4 insulating those areas needed to be etched. For this etch step, a thick positive photoresist (S1827, MicroChem Corp., Westborough, MA, USA) was used to mask the whole wafer except the area directly over the contacting pads, and reactive ion etching (RIE) was used to etch through the Parylene AF4 to the copper below. The wafer was etched using a 200 W RIE oxygen plasma for 3 min. When the etch step was completed the wafer was then stripped of photoresist and given a final cleaning, which marked the end of cleanroom processing.

**Figure 3:**
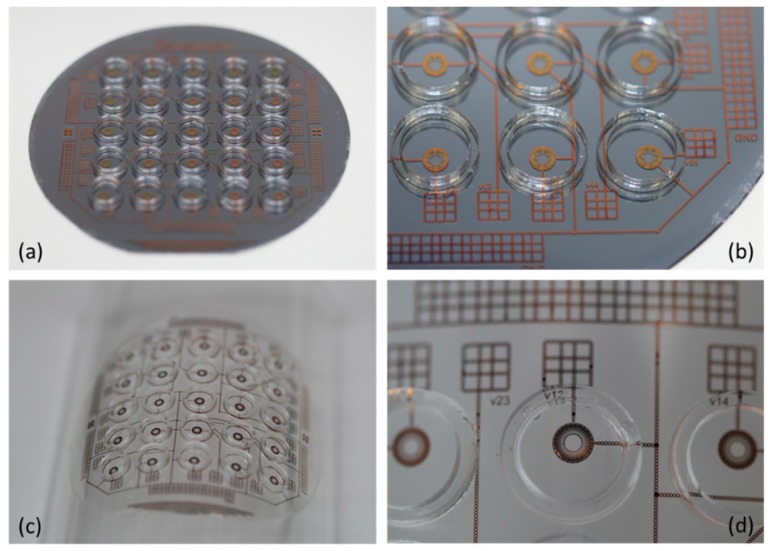
Photographs of (**a**) a 5 × 5 electrowetting on dielectric (EWOD) tunable lens array while still on a silicon carrier wafer used to ease the fabrication process, (**b**) a closer view of the lenses and contact pads of the array, (**c**) the PDMS sheet wrapped around a cylinder of a radius *r* = 70 mm, (**d**) a close-up view of a single, filled liquid lens in the array.

Final assembly was completed by affixing a lens chamber around each set of electrodes in the array in order to contain the liquids of the lens (see Figure 1b). For this, a 1.5-mm thick, flexible and transparent sheet composed of 50% ethylene vinyl acetate copolymer and 50% petroleum resin was cut into rings and then attached to the flexible substrate by placing the wafer on a 90 °C hotplate for 4 min. This melts the bottom layer of the rings and allows them to adhere to the flexible substrate. Finally, the substrate was carefully peeled from the carrier wafer, and the array of wells is ready to be filled with liquid to create the lenses. Each liquid lens was formed by manually placing a silicon oil droplet over the hydrophobic disk over the electrodes in the center of the well and then filling the surround area with water. The result, with water and oil pinned at the hydrophilic/hydrophobic interface can be seen in Figure 1b. After this, the liquids are sealed inside the lens chamber by adhering a polyethylene terephthalate (PET) cover to the top of each ring. The only remaining step was to attach wires to the contact pads, so that voltage can be applied to induce electrowetting actuation. This was done using a conductive, double sided tape. Photographs of the fully assembled flexible sheet can be seen in Figure 3, which exhibit a 5 × 5 array of lenses with a center-to-center spacing of 10mm between each of the 25 lenses in the square grid array. This 10-mm pitch allows for the space required to align CMOS image sensors beneath each lens of the array. Since we designed this lens to image far field objects, this 10-mm pitch only requires that the substrate be not wrapped on a surface of so small a radius that there is no longer overlap between the field of view of each individual lens in the array. However, in order to do imaging of close-range objects the array would need to be redesigned at a smaller pitch. In this case, very small form factor CMOS sensors would need to be used. 

## 3. Results

**Figure 4:**
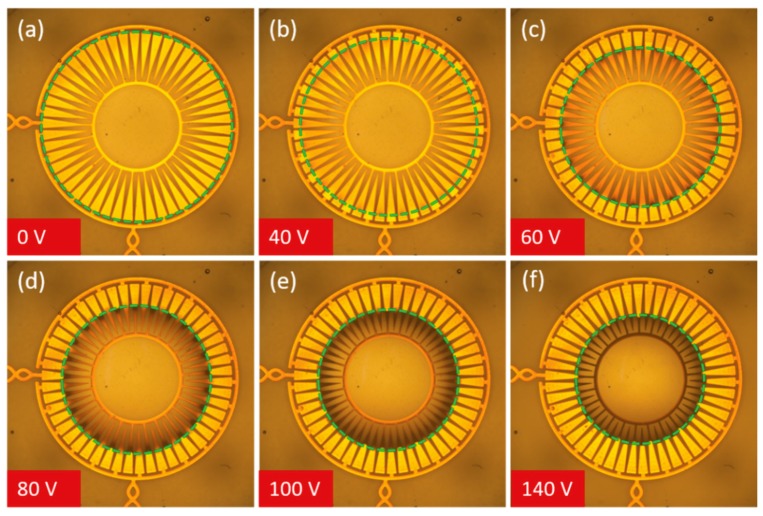
Images of a single lens during actuation, where all applied voltages are AC square waves at 10 kHz, all values peak-to-peak. The dashed green circles have been added to highlight the boundary between the oil droplet and the water that surrounds it. (**a**) The initial, rest state of the lens. With no applied voltage the oil droplet is pinned at the hydrophilic/hydrophobic boundary. (**b**) At 40 V, the oil droplet has begun to be squeezed as the water is attracted toward the underlying electrodes. This squeezing increases steadily with increased applied voltage as seen in (**c**) at 60 V, (**d**) at 80 V, (**e**) at 100 V and finally (**f**) at 140 V.

Once fabrication and assembly had been completed, individual lenses of the array were individually characterized before being used as an array. The first step was to examine how much actuation occurred within the lenses as voltage was applied across interdigitated electrodes. This actuation was first observed with the use of a microscope to measure how much the oil droplet was squeezed. These results can be seen in Figure 4. As expected, the oil droplet is initially pinned to the hydrophilic/hydrophobic boundary at the outer edge of the electrodes (see Figure 4a), but as increasing voltage is applied the water is attracted down and in towards the underlying electrodes, squeezing the oil droplet inwards. The measured current through the device also increased with increased applied voltage. Current values measured only a few μA at low voltages and reached a maximum of 20.77 mA (RMS) at our maximum driving voltage (150 V peak-to-peak).

**Figure 5:**
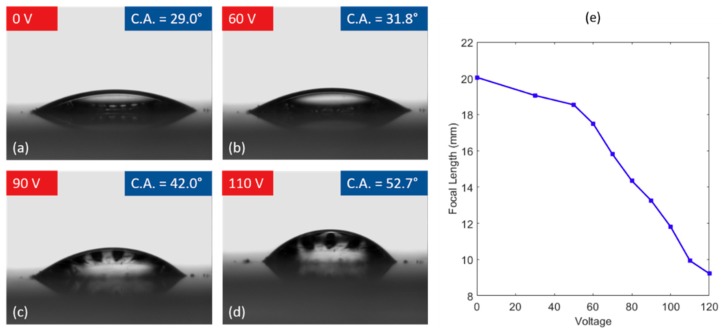
Goniometer images (profile view) of the lens formed by a silicone oil droplet surrounded by water (10 kHz AC square wave, all values peak-to-peak). (**a**) Initial rest state of lens with contact angle of 29.0° at 0 V. (**b**) The contact angle of the oil droplet increases to 31.8° at 60 V applied, corresponding to a decrease in the radius of curvature of the lens. (**c**) Contact angle of 42.0° at 90 V. (**d**) Contact angle of 52.7° at 110 V. (**e**) Plot of the focal length of the lens vs. the driving voltage (oil volume: 0.6253 μL).

In order to get more information about the change in shape of the oil droplet as voltage is applied we also observed the profile view of the lens. This was done using a goniometer (OCA 15+, DataPhysics Instruments Inc., Filderstadt, Germany), which allowed us to measure the change in contact angle with respect to voltage, as well as extract the surface profile of the lens at any given voltage. From the contact angle we can observe radius of curvature of our lens with respect to voltage and use this information to directly compute the focal length of the lens. These goniometer results can be seen in Figure 5, where we observed a change in the contact angle from 29.0° to 52.7° for a range of voltages from 0 V to 110 V. From the goniometer and microscope results, we can also find the range of focal length for our lenses. Since silicone oil has a higher index of refraction than water, the lens will always have a positive focal length, which shortens as increasing voltage is applied. We found such focal length range to be from 20.2 mm to 9.2 mm.

**Figure 6:**
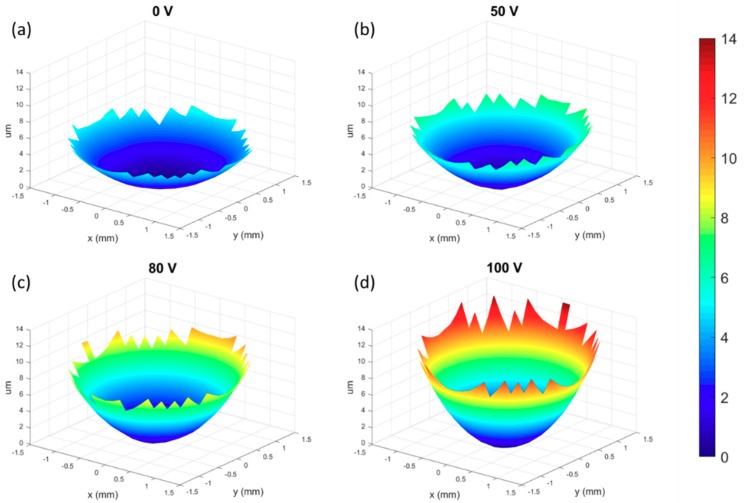
Plots generated by a Shack–Hartmann wavefront sensor showing the shape of a plane wave (from a 5mA HeNe laser) after it has passed through the lens under different levels of actuation. The *z*-scale and corresponding color bar units are in μm (10 kHz AC square wave, all values peak-to-peak). (**a**) Initial wavefront shape with 0 V applied; (**b**) 50 V applied; (**c**) 80 V applied; (**d**) 100 V applied.

While the goniometer allowed us to observe a 2D surface profile of the liquid lens shape during electrowetting, we were able to acquire more complete, 3D information about the lens shape by using a Shack–Hartman wavefront sensor (SHWS) [32,33]. A SHWS consists of a dense, uniform microlens array and a digital detector. When a plane wave impinges squarely on the SHWS, each microlens focuses this planewave into a point in the center of its cell. When a wavefront of a different shape (for instance a spherical wavefront) impinges on the SHWS the angle of the wave in a small area can be determined by measuring the displacement of the focal spot from the center. This data can be reconstructed across the whole sensor to make a measurement of the shape of the wavefront. In Figure 6 we can again see that the effective focal length of the lens shortens with increasing applied voltage. 

The wavefront data collected by the SHWS (WFS150C, Thorlabs Inc., Newton, NJ, USA) was also fit to the Zernike polynomials, which are a radial basis set often used to describe wavefronts. This allows the wavefront to be decomposed into Zernike modes, which allows for description of wave aberration functions. We fit the data collected by the SHWS to the orthonormal Zernike functions up to the fourth order, which produces the coefficients of each Zernike mode, each of which corresponding to a different type of optical aberration [34]. This information can be seen in Table 1. It should be noted that the fit for determining Zernike modes was very good for all measurements, with mean fit errors on the order of 1 × 10^−11^ arcmin or lower and standard deviations fit errors on the order of 1 × 10^−^^4^ arcmin. The wavefront sensor data was then fed into ZEMAX, which was used to simulate the modulation transfer function (MTF) of the lens according to the method in [32]. The value of the MTF at 50% is reported in Table 2 along with the focal length of the lens. As expected, the MTF-50 value increases as the focal length decreases and the f-number (f/#) of the lens increases.

**Figure 7:**
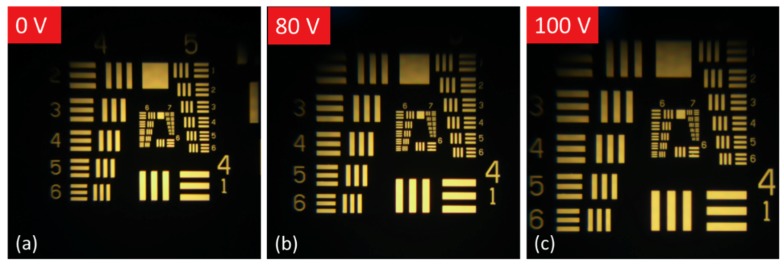
Images produced by a lens at different levels of actuation. As increasing voltage is applied the radius of curvature of the liquid lens decreases. Since the object distance remains fixed, the image produced is magnified with decreasing focal length, as expected. (**a**) Microscope image produced by focusing the microscope to the image plane produced by the convex lens at its initial rest state of 0 V applied. (**b**) 80 V is applied to the lens and the microscope focus is readjusted to match the new image plane of the lens, resulting in a larger image of the object. (**c**) When 100 V is applied to drive the lens, the microscope focus is again readjusted to produce a still-larger image.

From our SWHS analysis, we expect to be able to form a good quality image with the liquid lenses of our array. To directly observe the imaging capabilities of our lenses we first set up a simple imaging system on our microscope. This was done by placing an optical object (1951 USAF resolution target) on the stage of the microscope, and then raising the lens 4.42 mm above the object using spacers. The resulting virtual image (formed because the object was within the focal length of the lens) was then brought into focus through the microscope objective. As voltage was applied to the system the oil droplet was squeezed inwards, increasing the radius of curvature and shortening the focal length of the lens. After each change in applied voltage the focal plane of the microscope was adjusted to bring the image formed by the lens back into focus. These images can be seen in Figure 7. This shortening of the focal length corresponds to an increasing image distance. Since the object distance does not change, this results in the magnification of the resulting image as applied voltage increases.

**Figure 8:**
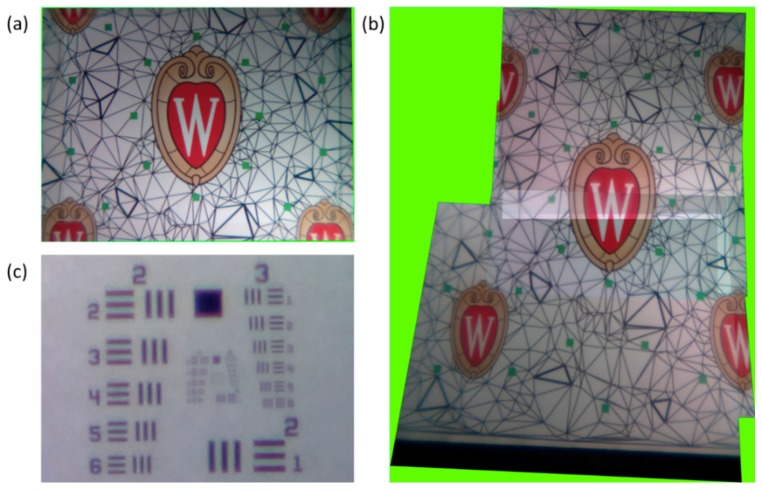
(**a**) A stitched image composed of nine separate images from the central 3 × 3 section of the array while the flexible sheet is on a flat surface. When on a flat surface the system has an FOV of 21.5°. (**b**) A stitched image composed from images obtained from the same central 3 × 3 section of the array, only now wrapped around a curved surface (radius equal to 70 mm). The FOV is increased significantly in the direction of curvature, up to 40.4°. (**c**) An image of a 1951-USAF resolution target obtained from one of the lenses in the array. Smallest resolvable line pair is group 3, element 6, corresponding to 14.25-line pairs/mm, or an angular resolution of 0.7 mrad. Similar results were obtained for other lenses in the array.

The final step in the process was to test the functionality of the array of lenses on our flexible sheet. In order to demonstrate improvement in the potential FOV of our system we obtained images from the central 3 × 3 lenses of the sheet when it was both flat and when it was wrapped on a convex curve, respectively. The setup used to obtain such images was constructed on an optical table and was composed of four basic components: a large optical object, in this case a printed scene including several crests; the flexible sheet containing the liquid lenses; an opaque, rigid sheet with an array of 1mm apertures over which the lenses of the flexible sheet were centered; and a small CMOS image sensor mounted on a 3-axis stage for precise positioning required to obtain images from each lens in the array. We used stereolithography (3D printing) to produce two different structures that contained the array of apertures. The first was flat and the second was curved, conforming to part of a cylinder with a radius of curvature of 70 mm. The optical object was placed 87 cm away from the flexible sheet of lenses. Images were first obtained by the nine lenses in the flexible sheet while it was laid over the flat aperture array. These images were then stitched together into a single image using the computer vision toolbox in MATLAB. The resulting image is shown in Figure 8a and has a FOV of 21.5°. Next, the flexible sheet of lenses was wrapped around the curved aperture array and images were again obtained from all 9 lenses. The same process was used to stitch these images together, the result of which can be seen in Figure 8b. The FOV increased significantly, up to 40.4°.

Using this same setup, we also obtained the resolution of our system. This was done using a standard 1951-U.S. Air Force target as the optical object, as shown in Figure 8c, yielding an angular resolution of 0.7 milliradians. This is only slightly below the theoretical resolution limit for the system of 0.61 mrad, approximated by the Rayleigh criterion for a circular aperture of a diameter equal to 1 mm and a wavelength of 500 nm. There are a number of possible sources of aberrations that lead to this decrease in resolution, among which is the tendency of liquid lenses to exhibit spherical aberration, as discussed earlier, and the possibility of contamination of the electrowetting surface by dust particles, which can lead to small asymmetries in lens actuation. This dust contamination can occur during the final filling of the lenses with the two immiscible liquids, which takes place outside of the cleanroom environment. Other potential sources of aberrations include the effect of the interdigitated electrodes, which squeeze the oil directly over the copper electrodes more strongly than the oil over the gaps in between. This leads to a slight scalloping effect at the edges while the lens is being actuated, which may introduce small aberrations. 

## 4. Discussion

We have detailed a successful fabrication method for constructing a 5 × 5 array of independently tunable, EWOD microlenses on a flexible sheet. This method improved on previous designs by changing materials to allow for a more stable hydrophobic/hydrophilic boundary necessary to define the rest state of the lens, and by reducing the thickness of the dielectric layer, which allowed for increased amount of actuation at applied voltages. The quality of our dielectric layer ensured that all lenses were robust against dielectric breakdown at voltages of 120 V or less, and many were robust at even greater voltages. When breakdown did occur, it was usually due to shorts through pinhole defects in the dielectric, occurring at high voltages and leading to bubbling in the lens. This actuation was observed and characterized using a variety of methods, including a top-down view of the change in oil droplet shape on the microscope, and a profile view of the change in contact angle as a function of applied voltage captured on a goniometer.

Characterization of the optical properties of our microlenses incorporated measurements with a SHWS to collect wavefront data in order to assess the shape of the lens. This data was then fit with Zernike polynomials. Spherical aberration is the most consistent and pronounced source of aberrations for our lenses and is a common characteristic of liquid lenses. However, the fact that only the central section of our liquid lens is utilized for imaging (the region where there are electrodes in the optical path) helped to minimize this aberration. We also conducted a preliminary verification of focal length tuning by observing the magnification of an image formed by the lens at close range to the object and again captured on the microscope. 

Finally, we demonstrated the functionality of a 3 × 3 section of our array on both a planar surface and on a cylindrical one. This testing was performed with a large object at distance (on the order of 1 m). Images were acquired from each of the nine lenses in the array for each configuration and then stitched into a single, larger image allowing us to demonstrate improvements in the FOV, nearly doubling on our cylindrical surface as compared to planar. Additionally, since there was more than sufficient overlap between all images in the array for stitching, even in the cylindrical arrangement, future work might see even larger improvements in FOV for the flexible sheet. Other future work on the flexible array includes further refinement of the fabrication process in order to verify consistency and reproducibility of lens quality as measured by resolution and Zernike coefficients. This would involve more detailed measurements of the resolution of both individual lenses and of the system as a whole. Other valuable characterizations of the lens would involve assessing the degree of chromatic aberration and making experimental measurements of response times and focal length repeatability of the lens. Additionally, we might dive into further exploration of different configurations of the flexible array, such as on a concave surface to explore its 3D imaging capabilities. Utilizing the array to imagine scenes with greater depth of field should also be explored, since the independent tunability of the focal length of each lens in the array will allow us to capture images from multiple focal planes simultaneously. 

## Figures and Tables

**Table 1:** Zernike coefficients (in μm) of our lens at different applied voltages, corresponding to the wavefronts shown in Figure 6. Note that n refers to the radial order of the polynomial and m refers to angular frequency.

Index ^1^	(n,m)	Mode name	Zernike Coefficients			
			0 V	50 V	80 V	100 V
0	(0,0)	Piston	−1.418	−2.605	−2.903	−3.966
1	(1,−1)	Tilt y	0.024	0.164	−0.214	−0.729
2	(1,1)	Tilt x	−0.061	0.171	0.247	1.001
3	(2,−2)	Astigmatism ±45°	−0.05	0.076	0.026	−0.032
4	(2,0)	Defocus/Power	1.525	2.093	2.777	3.675
5	(2,2)	Astigmatism 0/90°	0.006	0.060	0.011	−0.012
7	(3,−1)	Coma x	−0.004	0.000	0.016	0.033
8	(3,1)	Coma y	−0.029	−0.001	−0.029	−0.031
12	(4,0)	Spherical	0.034	0.013	0.063	0.100

^1^ Optical Society of America (OSA)/American National Standards Institute (ANSI) index numbering for Zernike modes.

**Table 2:** Measured focal length of the lens and MTF-50 values simulated in ZEMAX from Shack–Hartmann wavefront sensor data reported in Figure 6 and Table 1.

	0 V	50 V	80 V	100 V
**Focal length (mm)**	20.30	17.27	12.39	9.62
**Simulated MTF-50 (cycles/mm)**	15	34	35	56
